# Supplementary material for: Transcriptomics Analysis of Lens from Patients with Posterior Subcapsular Congenital Cataract
Source: Genes (Basel). 2021 Nov 27;12(12):1904. doi: 10.3390/genes12121904 (PMC8702110; doi:10.3390/genes12121904)
Supplement: Supplementary file 1 [file genes-12-01904-s001.zip › genes-1449240-supplementary.pdf]

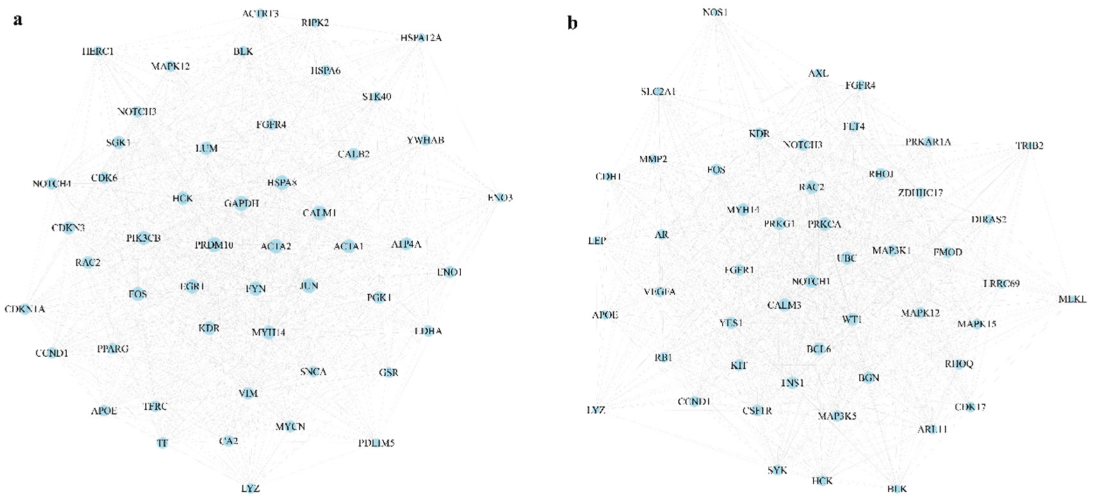

Figure S1. The top 50 PPI networks for differentially expressed proteins in lens epiehelial cells (**a**) and lens fiber cells (**b**).

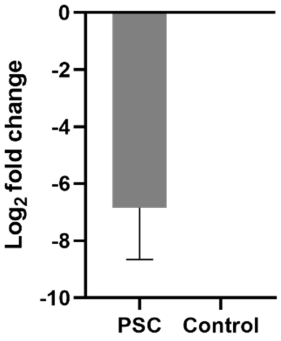

Figure S2. Relative mRNA expression in anterior lens capsules from posterior subcapsular congenital cataract patients and normal controls. The results showed that the mRNA expression of HTRA1 was significantly downregulated in posterior congenital cataract patients compared with normal controls, and the mean fold change was 158.62 ($p < 0.0001$).
